# Intraspecific Variation in Nickel Tolerance and Hyperaccumulation among Serpentine and Limestone Populations of *Odontarrhena serpyllifolia* (Brassicaceae: Alysseae) from the Iberian Peninsula

**DOI:** 10.3390/plants10040800

**Published:** 2021-04-19

**Authors:** A. Joseph Pollard, Grace L. McCartha, Celestino Quintela-Sabarís, Thomas A. Flynn, Maria K. Sobczyk, J. Andrew C. Smith

**Affiliations:** 1 Department of Biology, Furman University, Greenville, SC 29613, USA; grace.mccartha@smail.astate.edu; 2 Department of Biological Sciences, Arkansas State University, Jonesboro, AR 72401, USA; 3 Department of Soil Science and Agricultural Chemistry, University of Santiago de Compostela, CP 15782 Santiago de Compostela, Spain; tino.quintela.sabaris@gmail.com; 4 Department of Plant Sciences, University of Oxford, Oxford OX1 3RB, UK; t.flynn@bsg-ecology.com (T.A.F.); maria.sobczyk@bristol.ac.uk (M.K.S.); 5 BSG Ecology Ltd., Worton Park, Worton OX29 4SX, UK; 6 MRC Integrative Epidemiology Unit, Bristol Medical School, University of Bristol, Bristol BS8 2BN, UK

**Keywords:** ultramafic, serpentine, nickel, hyperaccumulation, facultative hyperaccumulator, metal tolerance, *Odontarrhena*, *Alyssum*

## Abstract

*Odontarrhena serpyllifolia* (Desf.) Jord. & Fourr. (=*Alyssum serpyllifolium* Desf.) occurs in the Iberian Peninsula and adjacent areas on a variety of soils including both limestone and serpentine (ultramafic) substrates. Populations endemic to serpentine are known to hyperaccumulate nickel, and on account of this remarkable phenotype have, at times, been proposed for recognition as taxonomically distinct subspecies or even species. It remains unclear, however, to what extent variation in nickel hyperaccumulation within this taxon merely reflects differences in the substrate, or whether the different populations show local adaptation to their particular habitats. To help clarify the physiological basis of variation in nickel hyperaccumulation among these populations, 3 serpentine accessions and 3 limestone accessions were cultivated hydroponically under common-garden conditions incorporating a range of Ni concentrations, along with 2 closely related non-accumulator species, *Clypeola jonthlaspi* L. and *Alyssum montanum* L. As a group, serpentine accessions of *O. serpyllifolia* were able to tolerate Ni concentrations approximately 10-fold higher than limestone accessions, but a continuous spectrum of Ni tolerance was observed among populations, with the least tolerant serpentine accession not being significantly different from the most tolerant limestone accession. Serpentine accessions maintained relatively constant tissue concentrations of Ca, Mg, K, and Fe across the whole range of Ni exposures, whereas in the limestone accessions, these elements fluctuated widely in response to Ni toxicity. Hyperaccumulation of Ni, defined here as foliar Ni concentrations exceeding 1g kg^−1^ of dry biomass in plants not showing significant growth reduction, occurred in all accessions of *O. serpyllifolia*, but the higher Ni tolerance of serpentine accessions allowed them to hyperaccumulate more strongly. Of the reference species, *C. jonthlaspi* responded similarly to the limestone accessions of *O. serpyllifolia*, whereas *A. montanum* displayed by far the lowest degree of Ni tolerance and exhibited low foliar Ni concentrations, which only exceeded 1 g kg^−1^ in plants showing severe Ni toxicity. The continuous spectrum of physiological responses among these accessions does not lend support to segregation of the serpentine populations of *O. serpyllifolia* as distinct species. However, the pronounced differences in degrees of Ni tolerance, hyperaccumulation, and elemental homeostasis observed among these accessions under common-garden conditions argues for the existence of population-level adaptation to their local substrates.

## 1. Introduction

Hyperaccumulators are plants that take up and store in their shoots exceptionally high concentrations of elements typically found in trace quantities in soils and biological materials [1]. Hyperaccumulation of heavy metals and metalloids is a globally rare phenomenon, documented in approximately 700 plant species, of which almost 75% are hyperaccumulators of nickel (Ni) [2,3]. Nickel hyperaccumulation occurs most commonly on serpentine soils, i.e., those derived from weathering of ultramafic rocks, which are typically enriched in Ni along with iron (Fe), magnesium (Mg), chromium (Cr), and cobalt (Co) [4,5,6]. Because of their unusual physico-chemical properties, serpentine soils are inhospitable to most plants, and often harbor a flora containing a high proportion of endemic species [4,7,8,9,10].

Most hyperaccumulators are restricted to metalliferous soils and are thus termed obligate metallophytes; however, about 10–15% of hyperaccumulator species are facultative metallophytes, meaning that they occur on both metalliferous and normal soils [11,12]. These facultative hyperaccumulators include some of the best-studied model systems for research on hyperaccumulation, including *Noccaea caerulescens* (J.Presl & C.Presl) F.K.Mey. (=*Thlaspi caerulescens* J.Presl & C.Presl) and *Arabidopsis halleri* (L.) O’Kane & Al-Shehbaz. One of the advantages of studying facultative hyperaccumulators is that they allow comparisons of intraspecific variation in physiology, genetics, and ecology among conspecific populations from metalliferous and non-metalliferous soils [11].

The physiological mechanisms underlying hyperaccumulation have been intensively studied and current knowledge is summarized in recent reviews representing many perspectives, including genetic, molecular, and evolutionary biology [13,14,15,16,17,18,19,20]. In very broad terms, hyperaccumulation involves the two suites of processes that can be termed metal accumulation and metal tolerance. In other words, hyperaccumulators must possess mechanisms to take up, transport, and sequester the particular metal(s) in question, but must also possess the ability to survive exposure to high concentrations of potentially toxic metals in the soil and in their tissues. Tolerance and accumulation may share some mechanisms but are also at least partially under independent physiological and genetic control [21,22,23].

Defining the concepts of metal hyperaccumulation and tolerance can be problematic. For hyperaccumulation, most researchers have adopted standard threshold criteria. Historically, the first example of this approach was the proposal that hyperaccumulators of Ni be recognized based on foliar tissue concentrations exceeding 1000 µg Ni per gram of dry leaf matter [24,25], equivalent to 1.0 g kg^−1^ or 0.1% (*w*/*w*). Subsequently, hyperaccumulation criteria have been proposed for other metals, sometimes at lower or higher concentrations depending on the abundance of the respective elements in nature, e.g., 0.1 g kg^−1^ for cadmium and thallium, or 10 g kg^−1^ for manganese [1]. These thresholds are not arbitrary, as they were based on concentrations that are at least 1 order of magnitude higher than that found in most plants on metalliferous soils and 2–3 orders of magnitude higher than in plants on normal soils, although their absolute nature has received some criticism [1].

In contrast, metal tolerance (or hypertolerance) is an entirely relative term, and no attempts have been made to establish consistent criteria. Initial indications of metal tolerance are usually field observations that certain plants grow with relative vigor on metal-rich soil where few other species survive. Experimental investigations may compare survival, growth, or reproduction of putatively tolerant plants to a non-tolerant reference group, when cultivated in a uniform, metalliferous medium, such as homogenized field soil, potting soil amended with metal salts, or hydroponic culture solution [26]. The choice of the reference group can be critical because some species are inherently more sensitive than others. Choosing an appropriate metal concentration in the experimental medium is also important; too low a concentration may fail to suppress the reference species, but excessively high concentrations may be uniformly toxic [11]. Ideally, tolerant plants should be compared with several reference groups across a range of metal concentrations.

In the Mediterranean basin and southwestern Asia, a remarkable diversity of Ni-hyperaccumulating taxa occurs in the tribe Alysseae of the family Brassicaceae. Until recently, most of these hyperaccumulators were classified in *Alyssum* sect. *Odontarrhena* (C.A.Mey.) W.D.J.Koch. However, recent phylogenetic research [27,28,29,30,31] has shown that *Alyssum*, as traditionally circumscribed, is not monophyletic. In particular, the species in sect. *Odontarrena* are not closely related to *Alyssum* L. sect. *Alyssum*, but rather have a sister-group relationship to the genera *Clypeola* L. and *Meniocus* Desv. [29]. We follow here the taxonomic treatment recommended by Španiel et al. [31] and recognize *Odontarrhena* C.A.Mey. at a generic rank.

As thus construed, the genus *Odontarrhena* includes 40–50 species of Ni hyperaccumulators [28,32,33,34,35]. The majority are obligate metallophytes [36,37], but eight or more species are facultative hyperaccumulators [11]. *Odontarrhena serpyllifolia* (Desf.) Jord. & Fourr. (=*Alyssum serpyllifolium* Desf. [31]) occurs near the western limit of the distribution of the genus, in the Iberian Peninsula, plus small areas of France and Morocco [38]. Plants growing on serpentine soil in Portugal and Spain are known to hyperaccumulate Ni [33].

In addition to the debate over generic circumscription, there has also been controversy regarding species boundaries. Whether *O. serpyllifolia* is considered a facultative hyperaccumulator is highly dependent on the taxonomic treatment of this entity. The taxon is sufficiently variable across its range that some authors have proposed segregating the serpentine populations as distinct subspecies or species, including *Alyssum serpyllifolium* ssp. *lusitanicum* T.R.Dudley & P.Silva (=*A. pintodasilvae* T.R.Dudley [39]); *Alyssum serpyllifolium* ssp. *malacitanum* Rivas Goday (=*A. malacitanum* (Rivas Goday) T.R.Dudley [40]); and perhaps ‘*A guitianii*’, although it appears this name was never validly published [41]. However, this partitioning of the species has not been accepted by most of the recent floristic and systematic treatments [31,42,43], which instead treat the proposed serpentine-endemic segregates as synonyms of the species *O. serpyllifolia* (=*A. serpyllifolium*). Likewise, an intensive population-genetic study [44] found that genetic divergence between serpentine and non-serpentine accessions of *O. serpyllifolia* was no greater than among populations from a given soil type, concluding that there was no support for specific recognition.

Metal concentrations in plant tissues in the field result both from inherent properties of the plants—presumably under genetic control—and from differences in the soil environment, including concentration, chemical speciation, and solubility of metals. Understanding the contributions made by these genetic and environmental factors often requires cultivation of plants in a uniform and standardized medium, described as common-garden studies [45]. For facultative hyperaccumulators, such studies are essential to reveal whether low metal concentrations in plants growing on non-metalliferous sites result from low metal availability in the soil, physiological/genetic differences between plants, or some interaction of the two.

The purpose of this research was to characterize the variation in physiological responses that occur within a widespread, facultative hyperaccumulator species known to occur on a variety of substrates and to exhibit pronounced phenotypic heterogeneity across its distribution. Although *Odontarrhena* includes more known hyperaccumulating species than any other genus, the great majority are serpentine-endemic, obligate hyperaccumulators, often with very restricted geographic distributions. In contrast, this report describes the results of common-garden experiments on *O. serpyllifolia* accessions collected as seeds from populations distributed across the Iberian Peninsula, including both serpentine and limestone (calcareous) soils. Plants were grown in a uniform environment so that intraspecific variation, presumably the result of local genetic adaptation, could be separated from physiological and phenotypic plasticity. All the accessions were exposed to a range of Ni concentrations in hydroponic cultivation, while keeping the concentration of other nutrients constant, allowing assessment of (i) variation in Ni tolerance and toxicity among accessions, (ii) variation among accessions in ability to hyperaccumulate Ni, and (iii) effects of Ni on uptake and storage of other essential elements—potassium (K), magnesium (Mg), calcium (Ca), and iron (Fe)—compared among the different accessions of *O. serpyllifolia*.

As explained above, the experimental design included non-metallophyte reference species; however, because almost all *Odontarrhena* spp. are capable of hyperaccumulation [37], it was necessary to use species from other closely related genera in the tribe Alysseae. Two such species were employed, neither of which has been reported to be commonly associated with serpentine or other ultramafic soil [34,42,43,46]. *Alyssum montanum* L. has been used as a reference species in previous research, which concluded that it is nickel-intolerant and does not hyperaccumulate metals [47,48]; however, it should be noted that recent phylogenetic research suggests that *Alyssum sensu stricto* is not sister to *Odontarrhena*. Rather, the genus *Clypeola* L. is now considered part of the sister-group to *Odontarrhena* [29,30,31], so *C. jonthlaspi* L. was selected as a second reference species. Although hyperaccumulation may show a phylogenetic signal across lineages [28], it appears that no previous studies have characterized the responses of *C. jonthlaspi* to soil metals. Thus, a further objective of this research was to examine Ni tolerance and accumulation in this species.

## 2. Results

### 2.1. Characteristics of Field Sites and Populations

**Figure 1:**
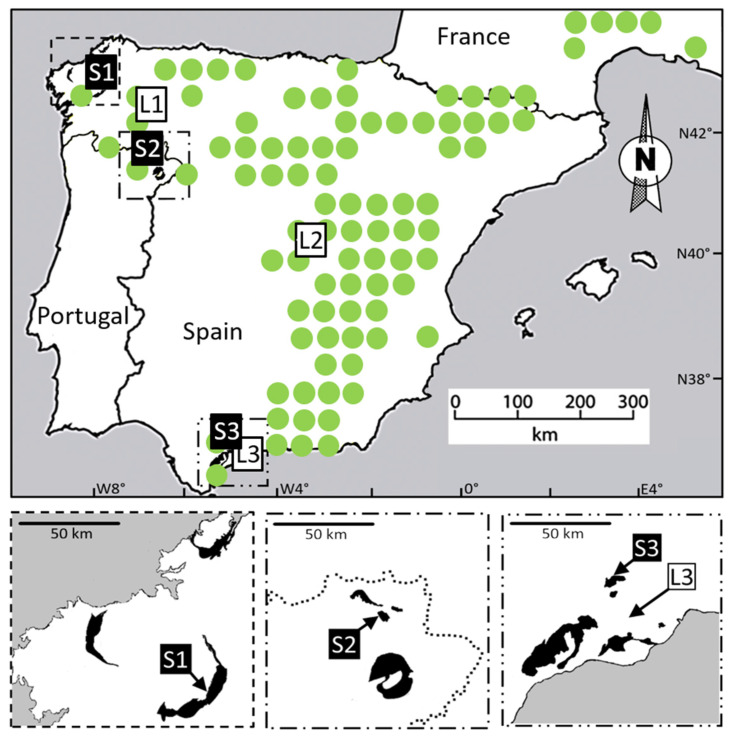
Outline map of the Iberian Peninsula showing approximate extent of serpentine areas (black) and collection sites for *Odontarrhena serpyllifolia* (numbered squares). Green dots indicate approximate Iberian range of *O. serpyllifolia* based on Atlas Florae Europaeae [38]. Sites S1, S2, and S3 were on serpentine substrates; sites L1, L2, and L3 were on limestone-derived soils. Inset panels below main map show enlarged outline of serpentine outcrops (mapping of serpentine based on [49,50]). See Table 1 for further details on sites.

Samples of *Odontarrhena serpyllifolia* were collected from five sites in Spain and one in Portugal (Table 1; Figure 1). The serpentine sites designated S1 in northwestern Spain and S2 in northeastern Portugal are separated by approximately 150 km. Limestone site L1 is situated in a Jurassic limestone gorge and lies roughly halfway between the two serpentine sites S1 and S2. Sites S3 and L3 are in the extreme south of Spain, about 750 km distant from the three sites previously described. The two southern sites are separated from each other by only 30 km; however, L3 is on calcareous soil in the Sierra de Mijas, whereas S3 is on serpentine soil in the nearby Sierra de Aguas. Site L2, in central Spain, is on limestone-derived soil, at least 300 km away from any of the other populations in this study, and a similar distance from any ultramafic outcrops.

Nickel concentrations in both soils and leaves of *O. serpyllifolia* sampled in the field were about two orders of magnitude higher at the serpentine sites than the limestone sites (Table 2). In all three serpentine accessions of *O. serpyllifolia*, mean foliar Ni concentration exceeded the hyperaccumulation criterion of 1.0 g kg^−1^. However, in accessions S1 and S2, a particularly wide range of foliar Ni concentrations was observed in the field material, with some individual plants showing values well below 1.0 g kg^−1^.

The two reference species, *Alyssum montanum* (Am) and *Clypeola jonthlaspi* (Cj), were grown from commercially supplied seeds (Table 1). Thus, the exact provenance of the populations was unknown, and we have no data on the characteristics of soils and plants in the field. Further information on seed sources is in the Materials and Methods.

### 2.2. Growth Differences among Accessions in Low-Nickel Solution

**Figure 2:**
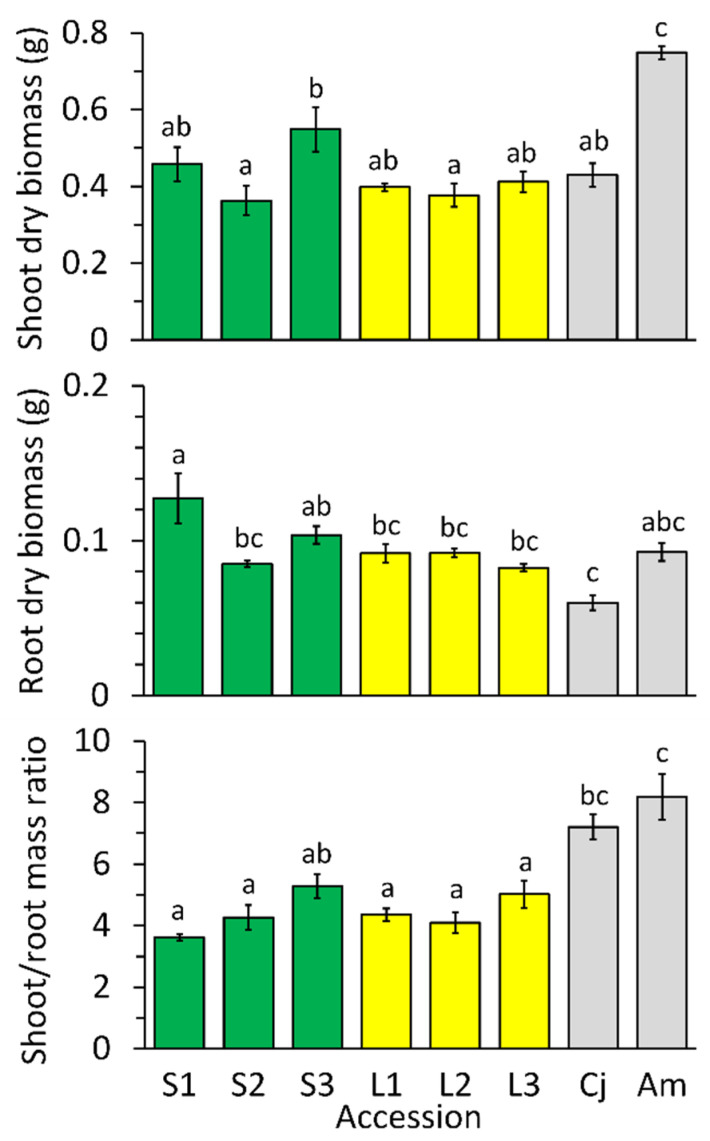
Mean dry biomass of shoots and roots, and shoot/root mass ratio, for plants grown in the “control” (0.1 µM Ni) hydroponic solution. Accessions S1–S3 (green bars) are *Odontarrhena serpyllifolia* from serpentine soils, whereas accessions L1–L3 (yellow bars) are *O. serpyllifolia* from limestone soil. Gray bars represent reference species used for comparison: *Clypeola jonthlaspi* (Cj) and *Alyssum montanum* (Am). Error bars indicate ± standard error of the mean. Lowercase letters on bars show significant differences between means according to Tukey’s multiple comparison procedure; means with different letters are significantly different at α = 0.05.

When grown in the control solution (Ni at micronutrient concentration of 0.1 µM), the eight accessions varied in mean shoot mass, mean root mass, and shoot/root mass ratio (Figure 2). Differences among accessions were statistically significant according to analysis of variance (shoot mass *F*_7,16_ = 13.24, *p <* 0.0001; root mass *F*_7,16_ = 7.18, *p <* 0.0006; shoot/root ratio *F*_7,16_ = 14.92, *p <* 0.0001). Conspicuously, the annual species *A. montanum* and *C. jonthlaspi* had significantly higher shoot/root ratios than the perennial *O. serpyllifolia*, indicating greater allocation to above-ground biomass in the annuals.

Inherent differences in plant size and biomass allocation can confound measurement of plant responses to metal toxicity [51]. To avoid this problem, analyses of Ni tolerance were based on an “Index of Tolerance” [52,53], derived by dividing each plant’s mass by the mean mass of the control (0.1 µM) group for that accession, multiplied by 100, to express growth in Ni solutions as a percentage of control growth.

Compared to the differences between species, intraspecific variation in shoot mass, root mass, and biomass allocation among the *O. serpyllifolia* accessions in the control solution was relatively small, with no consistent differences between plants from limestone and serpentine substrates. Independent-sample *t*-tests comparing the pooled means of limestone and serpentine *O. serpyllifolia* accessions were non-significant for all three characters (shoot mass *t*_16_ = 1.59, *p =* 0.131; root mass *t*_16_ = 1.98, *p =* 0.065; shoot/root ratio *t*_16_ = 0.28, *p =* 0.785), indicating no consistent differences in plant size or allocation between plants from the two substrates when grown in a common environment.

### 2.3. Variation in Nickel Tolerance

**Figure 3:**
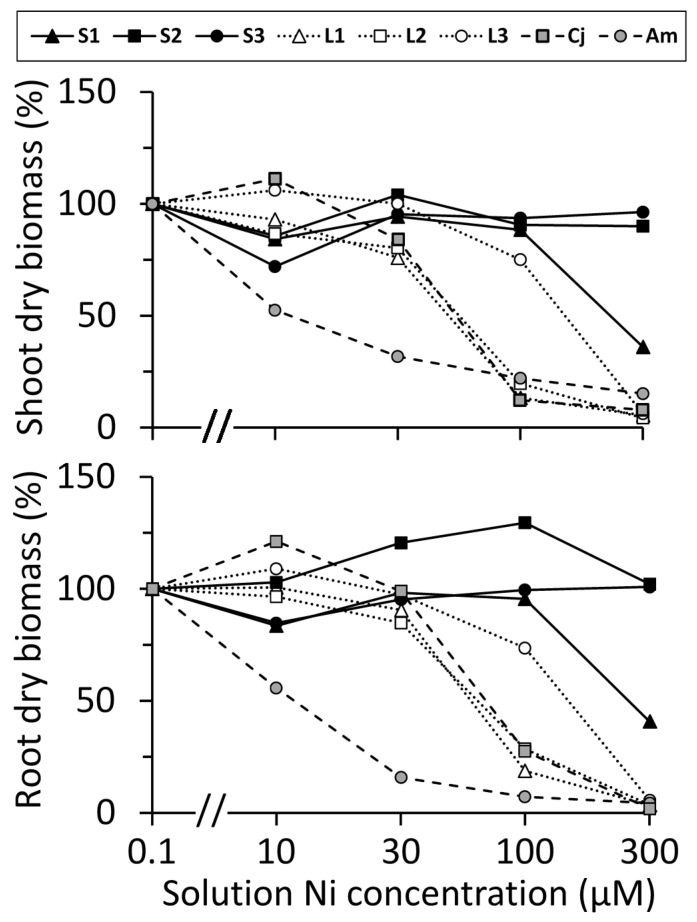
Root and shoot biomass of six accessions of *Odontarrhena serpyllifolia* (S1–S3 from serpentine soil; L1–L3 from limestone soil) plus the related species *Clypeola jonthlaspi* (Cj) and *Alyssum montanum* (Am), grown at five concentrations of NiSO_4_ in hydroponic culture solution. Plant biomass is expressed as a percentage of mass in the least concentrated solution. Solution Ni concentrations are plotted on a logarithmic scale.

Plant growth responses to Ni amendment of hydroponic media differed markedly among accessions (Figure 3). For each accession, root and shoot biomass production displayed similar trends of response to Ni. *Alyssum montanum* showed a severe reduction in the growth of roots and shoots at even the lowest Ni concentrations, with growth inhibition of approximately 50% at 10 µM Ni; this was a much greater sensitivity than found in any other accession. The three serpentine accessions of *O. serpyllifolia* exhibited generally greater Ni tolerance than the three limestone accessions. Serpentine accessions S2 and S3 were clearly the most tolerant, with no statistically significant reduction in growth of either roots or shoots in any treatment up to the highest Ni concentration tested (300 µM). All other groups showed some degree of statistically significant inhibition by Ni in the growth medium, as demonstrated by ANOVA (Table 3). Limestone accessions L1 and L2 were the most Ni-sensitive accessions of *O. serpyllifolia*, and *C. jonthlaspi* showed a very similar pattern of response to these accessions. The limestone accession L3 and serpentine accession S1 were intermediate in their sensitivity (Figure 3). Although some accessions had means greater than 100% in certain treatments, there was no statistically significant stimulation of growth by Ni in these experiments.

To quantify the different patterns of response seen among accessions, we used Dunnett’s multiple comparison procedure, a statistic that compares each treatment’s mean mass to the control (0.1 µM Ni) mean. In this way, we identified the mNOEC (maximum no observed effect concentration) for each population, which is the highest Ni concentration at which no statistically significant (α = 0.05) reduction in growth occurred (Table 4). Although there has been extensive discussion in the ecotoxicology literature about the appropriateness of the mNOEC concept for identifying “safe” concentrations of a toxicant [54,55], our use of mNOEC was merely a convenient metric for comparing the Ni sensitivity of different accessions in a standardized way. The sensitivity of *A. montanum* was reflected in its very low mNOEC value of 0.1 µM, as even the 10 µM Ni solution caused statistically significant reduction in growth of both roots and shoots of this species. At the opposite extreme, the mNOEC observed for accessions S2 and S3 of *O. serpyllifolia* was ≥300 µM, because even the highest Ni concentration employed in the experiment had no statistically significant effect on these plants. Further experiments would be necessary to quantify the limits of tolerance for these accessions. The other accessions had intermediate mNOEC values, in the range of 30–100 µM. Notably, serpentine accession S1, with mNOEC = 100 µM, was markedly less tolerant than S2 and S3. The mNOEC of *C. jonthlaspi* was 30 µM, a similar response to that of the limestone accessions of *O. serpyllifolia*.

### 2.4. Variation in Nickel Hyperaccumulation

**Figure 4:**
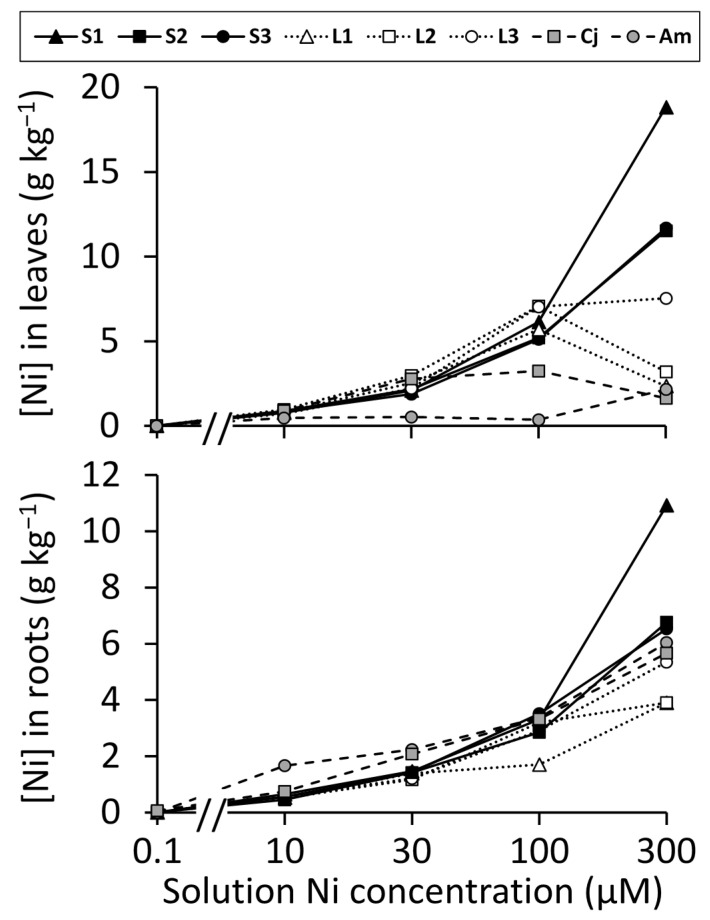
Nickel concentrations in root and leaf dry biomass in six accessions of *Odontarrhena serpyllifolia* (S1–S3 from serpentine soil; L1–L3 from limestone soil) plus the related species *Clypeola jonthlaspi* (Cj) and *Alyssum montanum* (Am), grown at five concentrations of NiSO_4_ in hydroponic culture solution. Solution Ni concentrations are plotted on a logarithmic scale.

Figure 4 shows the concentrations of Ni accumulated in leaves and roots of the hydroponically grown plants exposed to different Ni treatments. The roots of *A. montanum* accumulated Ni more strongly than those of other accessions in the 10 µM Ni solution. Statistical significance of this finding was confirmed by an ANOVA indicating significant differences among accessions at this concentration (*F*_7,10_ = 16.11, *p =* 0.0001), followed by Tukey’s multiple comparison test (α = 0.05), which showed a significant difference between *A. montanum* and all other plants, but no significant differences among the *O. serpyllifolia* accessions or *C. jonthlaspi*. Root Ni concentrations in the 30 µM Ni solution showed a similar pattern of significant differences (*F*_7,16_ = 4.33, *p =* 0.007), except *C. jonthlaspi* was intermediate and not significantly different from either *A. montanum* or *O. serpyllifolia*. At 100 µM Ni, there were few clear trends, although *O. serpyllifolia* accession L1 had significantly lower Ni accumulation in its roots than some other accessions (*F*_7,16_ = 4.57, *p =* 0.006). At 300 µM Ni, the only significant difference was that accession S1 had a higher Ni concentration in its roots than all other accessions (*F*_7,6_ = 20.81, *p* = 0.0008).

**Figure 5:**
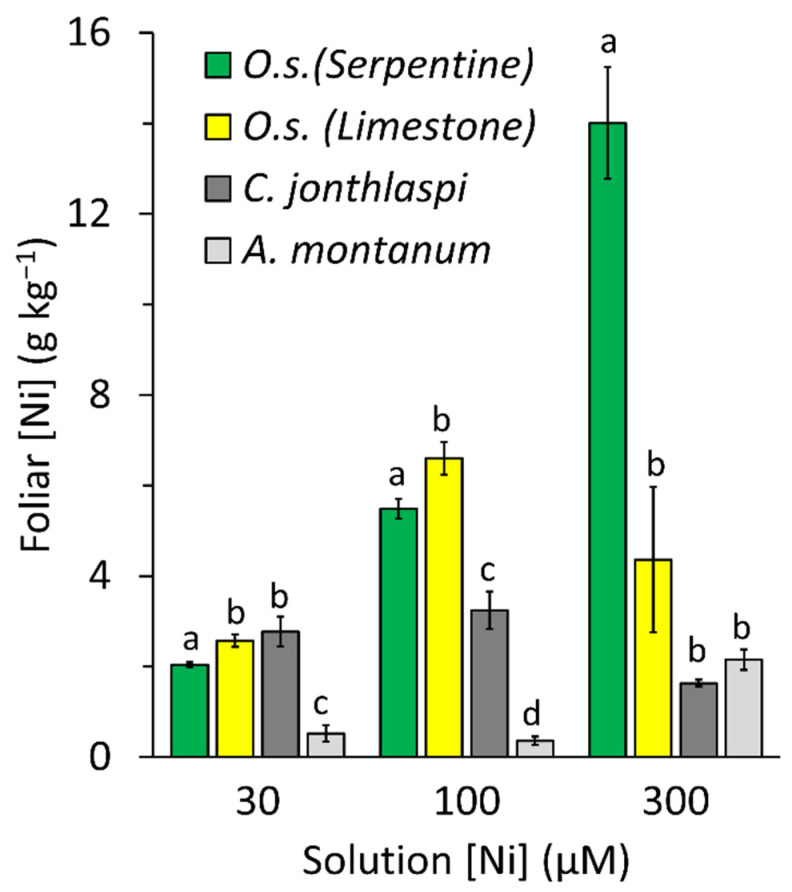
Mean foliar nickel concentration of plants grown in hydroponic solutions amended with NiSO_4_ at specified concentrations. The four plant groups include *Odontarrhena serpyllifolia* from three sites on serpentine soil, *O. serpyllifolia* from three sites on limestone soil, *Clypeola jonthlaspi*, and *Alyssum montanum*. Error bars represent ± standard error. Letters above bars indicate significant differences within a given cluster, based on Tukey’s multiple comparison procedure; means with different letters are significantly different at α = 0.05.

The patterns of Ni accumulation in leaves (Figure 4) were more complex. In hydroponic solutions containing 30 µM Ni and higher, all the *O. serpyllifolia* accessions and *C. jonthlaspi* had foliar Ni concentrations above the 1.0 g kg^−1^ threshold that defines hyperaccumulation. Specific comparisons among accessions are shown in Figure 5. The two lowest solution Ni concentrations are not shown in this figure, because foliar concentrations were all near zero in the 0.1 µM Ni treatment, whereas patterns of response in the 10 µM solution were like those at 30 µM Ni but below the hyperaccumulation threshold. To facilitate direct comparisons between substrates, *O. serpyllifolia* accessions were pooled into two groups representing serpentine and limestone sites. In the 30 µM Ni solution, the highest foliar Ni concentrations were observed in the limestone *O. serpyllifolia* accessions and *C. jonthlaspi*, which were not significantly different from each other according to Tukey’s multiple comparison test (α = 0.05). The serpentine *O. serpyllifolia* accessions had significantly lower Ni concentrations, though still above 2.0 g kg^−1^, whereas *A. montanum* had a mean Ni concentration that was significantly lower still and below 1.0 g kg^−1^. In the 100 µM Ni solution, the four groups were all significantly different from one another; highest foliar Ni concentrations were observed in the limestone *O. serpyllifolia* accessions, followed by the serpentine accessions of that species, followed by *C. jonthlaspi*. Once again, *A. montanum* had Ni concentrations below the level of hyperaccumulation. In the 300 µM Ni solution, the highest foliar Ni concentration by far was observed in the serpentine accessions of *O. serpyllifolia*, with a mean of approximately 14 g kg^−1^. There were no significant differences among the limestone *O. serpyllifolia*, *C. jonthlaspi*, and *A. montanum* groups, and all had mean Ni concentrations exceeding the hyperaccumulation threshold.

**Figure 6:**
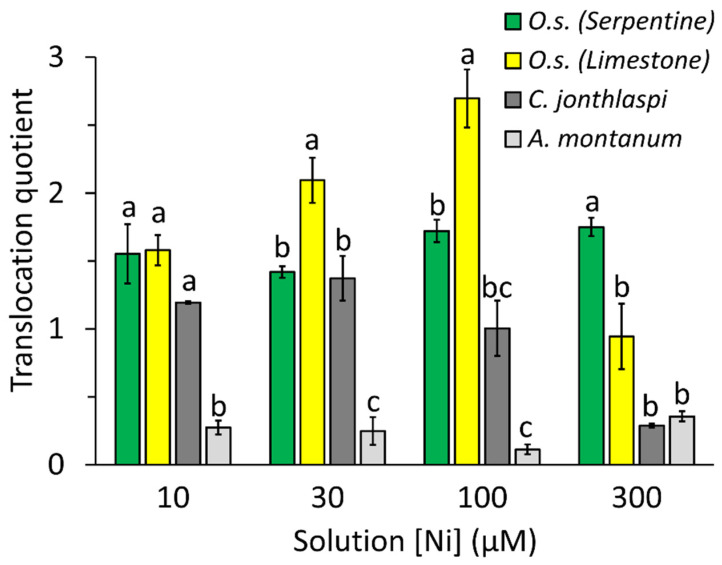
Translocation quotient (shoot Ni concentration/root Ni concentration on a dry-biomass basis) of plants grown in hydroponic solutions amended with NiSO_4_ at specified concentrations. The four plant groups include *Odontarrhena serpyllifolia* from three sites on serpentine soil, *O. serpyllifolia* from three sites on limestone soil, *Alyssum montanum*, and *Clypeola jonthlaspi*. Error bars represent ± standard error. Letters above bars indicate significant differences within a given cluster, based on Tukey’s multiple comparison procedure; means with different letters are significantly different at α = 0.05.

The translocation quotient (shoot concentration/root concentration) of trace elements is typically >1 for hyperaccumulators but <1 for non-accumulators [56]. Translocation quotients for Ni in the present study are shown in Figure 6. Data from the 0.1 µM Ni treatment are not shown, as they were extremely variable with large standard errors and no significant differences among accessions; presumably, this is because both root and shoot Ni concentrations were near zero, so the quotients were strongly affected by error variation. In all other solution concentrations, *A. montanum* had a Ni translocation quotient of 0.5 or less, indicating higher Ni concentrations in the roots than in the shoot. In contrast, the translocation quotient for serpentine accessions of *O. serpyllifolia* was consistently in the range from 1.4–1.8 across all treatments. Translocation quotients of the limestone *O. serpyllifolia* accessions varied: They were similar to those of the serpentine accessions in 10 µM Ni, significantly higher than the serpentine accessions at 30 and 100 µM Ni, and significantly lower than the serpentine accessions at 300 µM Ni (statistical significance based on Tukey’s test; see Figure 6). The translocation quotient for *C. jonthlaspi* was low in the 0.1 and 300 µM Ni solutions but exceeded 1.0 in intermediate solution concentrations.

### 2.5. Effects of Ni on Concentrations of Other Cations—Univariate Analyses

**Figure 7:**
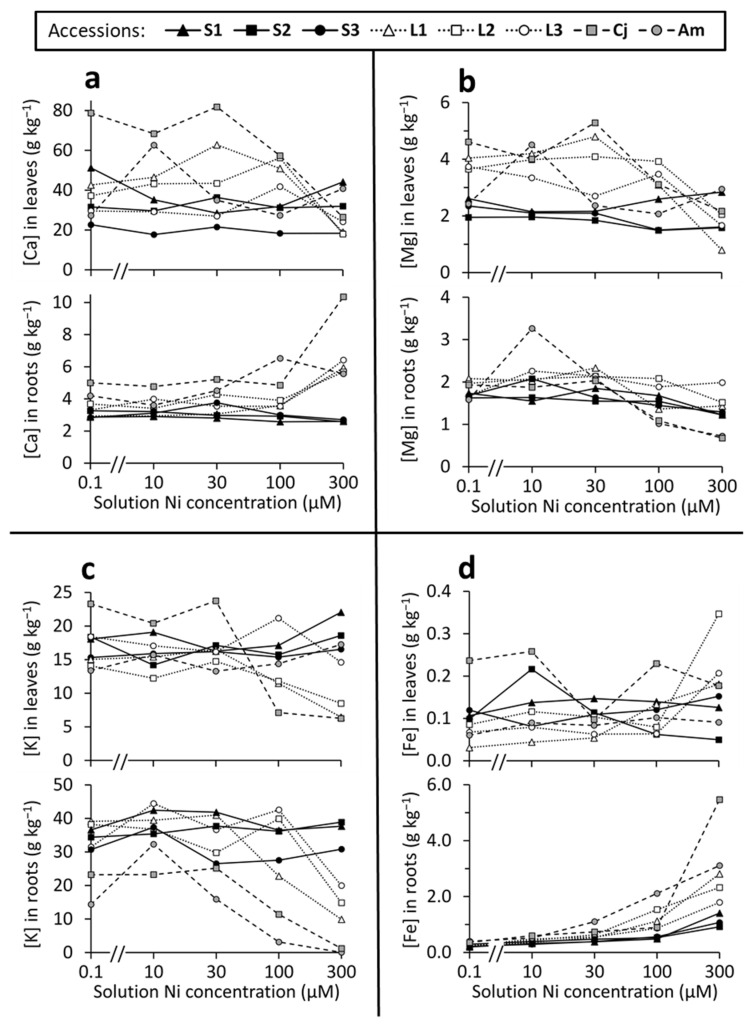
Elemental concentrations in roots and leaves of six accessions of *Odontarrhena serpyllifolia* (S1–S3 from serpentine soil; L1–L3 from limestone soil) plus the related species *Clypeola jonthlaspi* (Cj) and *Alyssum montanum* (Am), grown at 5 concentrations of NiSO_4_ in hydroponic culture. Solution Ni concentrations are plotted on a logarithmic scale. Elements are (**a**) calcium; (**b**) magnesium; (**c**) potassium; (**d**) iron.

Elemental analysis of Ca, Mg, K, and Fe in plant tissues (Figure 7) revealed complex patterns of variation, even though all plants were grown in hydroponic media made with identical concentrations of these elements, and only NiSO_4_ concentrations were varied. The summaries that follow will attempt to describe the most consistent patterns. For statistical analyses (Table 5), *O. serpyllifolia* accessions have been pooled by soil-type (serpentine vs. limestone), to simplify and generalize the results.

The concentration of Ca (Figure 7a) was, on average, about 10× higher in leaf biomass than in root biomass. In the serpentine accessions of *O. serpyllifolia*, both leaf and root Ca concentrations remained relatively constant with increasing Ni treatment, whereas the limestone accessions of *O. serpyllifolia*, along with both *A. montanum* and *C. jonthlaspi*, showed significant changes in both root and leaf Ca concentrations as solution Ni concentration increased (see ANOVA results in Table 5). In general, Ca concentrations in leaf tissues of non-serpentine plants decreased in solutions with higher Ni concentrations, whereas Ca concentrations in root tissues of these plants increased as solution Ni increased. These trends were most pronounced in the 300 µM Ni solution.

Concentrations of Mg (Figure 7b) were generally similar in root and leaf tissues. In the control solution, serpentine accessions of *O. serpyllifolia* were characterized by significantly lower leaf Mg concentrations than the limestone accessions (*t* test, *t*_16_ = 6.02; *p* < 0.0001). With increasing Ni concentration, both leaf and root Mg concentrations remained relatively constant in the serpentine accessions, although there was a small but statistically significant decline in root Mg concentration at the highest Ni concentrations (Table 5). For the limestone accessions of *O. serpyllifolia*, Mg concentrations in both leaves and roots were significantly reduced in the high-Ni growth solutions, with especially sharp decreases in shoot Mg at 300 µM Ni. A similar statistically significant decrease in Mg concentration was observed in high-Ni solutions for both leaves and roots of *C. jonthlaspi*, and for leaves of *A. montanum*. There was unexplained high variability in the root Mg concentrations of *A. montanum* (especially in the 10 µM Ni solution) and no statistically significant variation among treatments for this character.

Concentrations of K (Figure 7c) were approximately twice as high in root tissue as in leaves. For both leaf and root tissues, the serpentine accessions of *O. serpyllifolia* showed no statistically significant changes in K concentration in response to Ni in the growth medium. There was much greater variability in the other accessions, with statistically significant decreases in K concentration observed in the roots of limestone *O. serpyllifolia*, and in both leaves and roots of *C. jonthlaspi* and *A. montanum* at the highest Ni concentration. For the limestone *O. serpyllifolia* there was an apparent decrease in leaf K concentration of plants grown in 300 µM Ni solution, but it was not statistically significant.

Concentrations of Fe (Figure 7d) were, on average, approximately 10× higher in roots than in leaves. Leaf Fe concentration in serpentine *O. serpyllifolia* accessions, and also in *A. montanum*, did not vary significantly among Ni treatments (Table 5). The limestone accessions of *O. serpyllifolia* showed a statistically significant increase in foliar Fe concentration in solutions above 30 µM Ni. For *C. jonthlaspi*, there was also significant variation in foliar Fe concentration, but this was manifested primarily in an anomalously low Fe concentration in plants grown in the intermediate 30 µM Ni solution. In contrast to these highly variable results for leaves, the concentrations of Fe in root tissues responded to exogenous Ni in a remarkably consistent manner, with all accessions showing higher root Fe concentrations as Ni concentration in the growth medium increased. This trend was statistically significant for all accessions (Table 5), but the slope of the response was steepest in the reference species (*C. jonthlaspi* and *A. montanum*), intermediate in the limestone accessions of *O. serpyllifolia*, and least steep in the serpentine accessions of *O. serpyllifolia* (Figure 7d).

### 2.6. Effects of Ni on Concentrations of Other Cations—Multivariate Analysis

**Figure 8:**
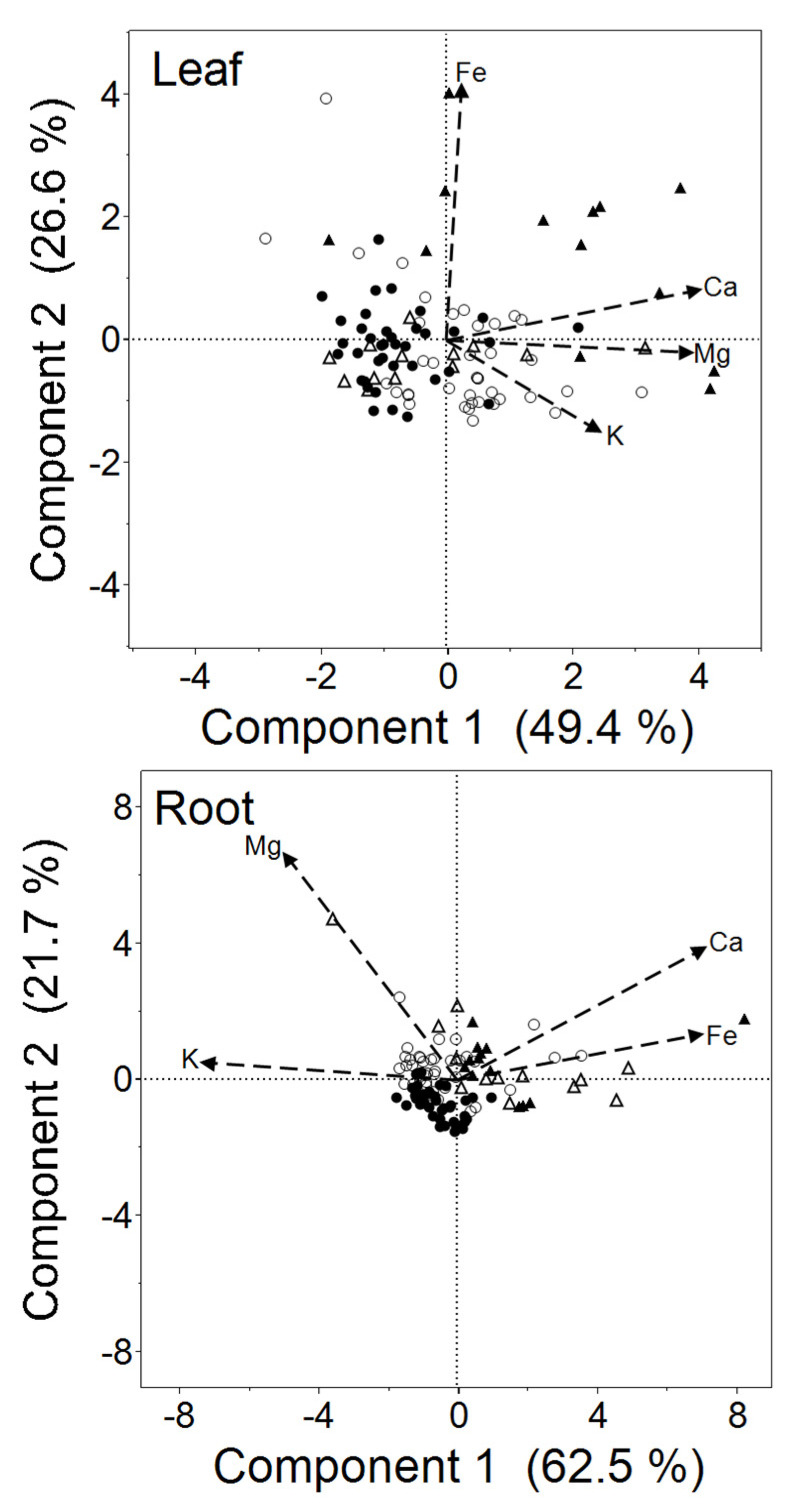
Biplots from principal components analysis of Ca, Mg, K, and Fe concentrations in plant tissues following hydroponic cultivation. Plant groups are *Odontarrhena serypyllifolia* from serpentine soil (black circles), *O. serypyllifolia* from limestone soil (open circles), *Clypeola jonthlaspi* (black triangles), and *Alyssum montanum* (open triangles). Upper panel shows results for leaves; lower panel shows results for roots. Eigenvectors indicating component loadings are shown as dashed arrows.

Principal components analysis (PCA) was used to examine correlated patterns of variation in Ca, Mg, K, and Fe among accessions. For leaves (Figure 8, upper panel), the first principal component accounted for 49.4% of total variation, and involved positive correlations in concentrations of Ca, Mg, and K. The second axis, accounting for 26.6% of variation, was affected most strongly by a positive loading for Fe, and to a lesser extent a negative loading for K. For roots (Figure 8, lower panel), the first principal component accounted for 62.5% of total variation, and involved positive loadings for Ca and Fe combined with negative loadings for K and Mg. The second PCA axis, accounting for 21.7% of variation, was affected most strongly by a positive loading for Mg and, to a lesser extent, for Ca.

Comparisons between serpentine *O. serpyllifolia,* limestone *O. serpyllifolia*, *C. jonthlaspi*, and *A. montanum* in their mean scores on the first two PCA axes were made by ANOVA. For leaves, there were significant differences on both axes (PCA1: *F*_3,100_ = 17.87, *p <* 0.0001; PCA2: *F*_3,100_ = 14.08, *p <* 0.0001). Tukey’s test indicated that *C. jonthlaspi* had significantly higher mean scores on both axes than any other groups. In addition, the limestone *O. serpyllifolia* had significantly higher scores on the first PCA axis than did the serpentine accessions of that species. All other pairwise comparisons were non-significant. For roots, there were again highly significant differences among the groups of accessions on both axes (PCA1: *F*_3,100_ = 12.58, *p <* 0.0001; PCA2: *F*_3,100_ = 17.88, *p <* 0.0001). Tukey’s test indicated that the accessions clustered into two groups along the first PCA axis, with *C. jonthlaspi* and *A. montanum* having higher mean scores than either the serpentine or limestone *O. serpyllifolia*. On the second PCA axis, there were no statistically significant differences among *C. jonthlaspi*, *A. montanum*, or the limestone *O. serpyllifolia*, but the serpentine accessions of *O. serpyllifolia* had a significantly lower mean score.

**Figure 9:**
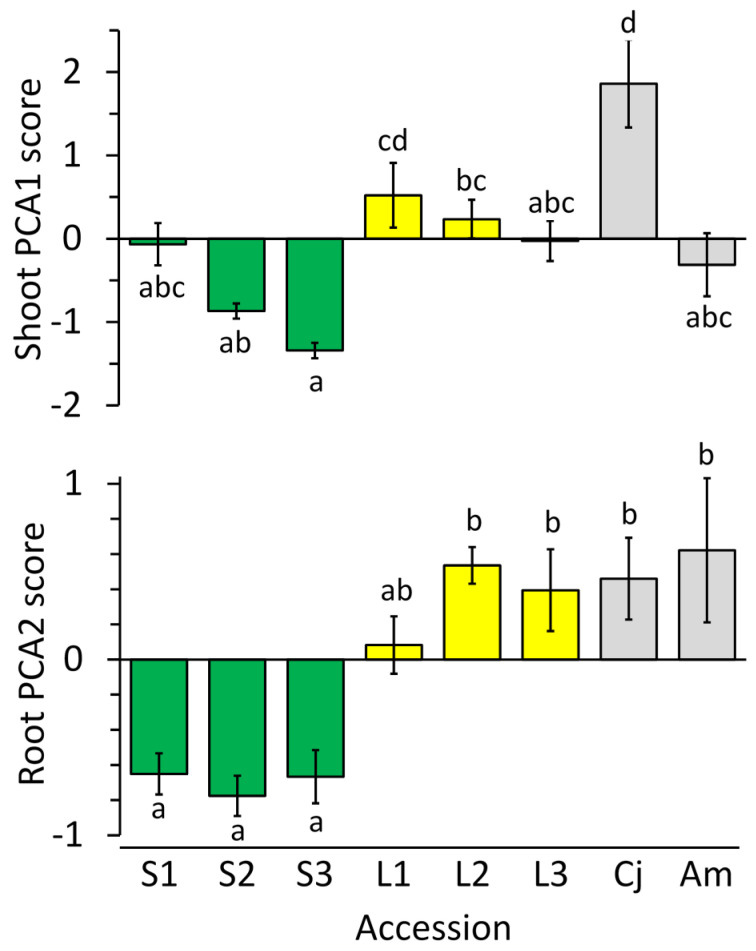
Mean scores from principal components analysis (PCA) of the concentration of Ca, Mg, K, and Fe in plant tissues. Upper panel shows scores on the first PCA axis for leaf tissues; lower panel shows scores on the second PCA axis for root tissues (see text for explanations of why these axes were selected for analysis). Accessions S1–S3 (green bars) are *Odontarrhena serpyllifolia* from serpentine soils, whereas accessions L1–L3 (yellow bars) are *O. serpyllifolia* from limestone soil; gray bars represent reference species *Clypeola jonthlaspi* (Cj) and *Alyssum montanum* (Am). Error bars indicate ± standard error of the mean. Lowercase letters on bars show significant differences between means according to Tukey’s multiple comparison procedure; means with different letters are significantly different at α = 0.05.

Because there appeared to be differentiation between serpentine and limestone accessions of *O. serpyllifolia* on some axes, specifically PCA1 for leaves and PCA2 for roots, we conducted more detailed ANOVAs comparing the individual accessions for these scores. For the leaves, the scores on the first PCA axis were generally higher for the limestone accessions, but there was a range of continuous variation (Figure 9, upper panel). Interestingly, accessions S1 and L3, which were previously identified as intermediate in their Ni-tolerance properties, were also intermediate in their elemental profile as indicated by scores on this axis. For root scores on the second PCA axis, there was again a spectrum of variation (Figure 9, lower panel). In this case, accession L1, which had not previously been recognized as intermediate, had a mean score that was not significantly different from any other accession.

The PCA summaries above are based on pooled results from all hydroponic Ni concentrations. To examine the effects of variation in exogenous Ni on the elemental profile of plant tissues, ANOVA was used to make comparisons of PCA scores among Ni treatments, separately for each category of plants. The results (Table 6) indicate that Ni treatment caused no statistically significant differences in elemental PCA scores of serpentine *O. serpyllifolia* leaves on either the first or second principal component axis, as was previously seen for individual element concentrations (Table 5). In roots of serpentine *O. serpyllifolia*, there were no significant differences in scores on the first PCA axis, but a highly significant difference on the second axis, with lower scores at 300 µM Ni, reflecting the lower Mg concentrations mentioned in the univariate analyses. Significant variation in PCA scores across Ni treatments was found for both leaves and roots of limestone *O. serpyllifolia* plants and *C. jonthlaspi* (Table 6). For *A. montanum*, there were significant differences among Ni treatments for scores on the first PCA axis for both leaves and roots, but not on the second axis.

## 3. Discussion

The overarching conclusions suggested by these results are that a spectrum of physiological ability to tolerate and accumulate Ni exists within *Odontarrhena serpyllifolia*, and that all the *O. serpyllifolia* accessions included in this study, whether collected from serpentine or limestone, can potentially hyperaccumulate Ni under appropriate conditions. This agrees with Quintela-Sabarís et al. [57], who reported that plants from some limestone populations of *O. serpyllifolia* were able to hyperaccumulate Ni when grown on compost-amended serpentine soils. However, these conclusions contradict the findings of three early studies [33,58,59], which reported that *O. serpyllifolia* plants from non-serpentine soil were highly sensitive to Ni toxicity and completely incapable of Ni hyperaccumulation. The provenance of the non-serpentine material was not specified in those early works, but it `appears that only a single non-serpentine accession was tested in each case, and possibly the same accession was used in all three. The contrasting conclusion presented here emphasizes the importance of more extensive sampling to reveal the full range of Ni tolerance and hyperaccumulation ability within the species.

Accessions of *O. serpyllifolia* from serpentine soils had higher Ni tolerance than those from limestone soils, in agreement with the hypothesis that elevated metal tolerance may evolve on substrates with high metal concentrations [11,60]. This pattern was not unexpected for a facultative metallophyte like *O. serpyllifolia*, with samples collected from both metalliferous and non-metalliferous soils. However, what has remained unresolved is the extent to which the wide range of phenotypes observed within *O. serpyllifolia sensu lato* can be explained by phenotypic plasticity or reflect local adaptation to contrasting edaphic environments. A powerful approach to discerning environmental vs. genetic and physiological sources of variation is to conduct common-garden experiments under controlled conditions under which the responses to specific factors—such as substrate Ni concentration—can be systematically explored, as performed in the present study.

As mentioned earlier, many common plants cannot grow well on serpentine outcrops. Several properties of serpentine soil may contribute to this, including a very high Mg:Ca ratio, low availability of macronutrients such as K and P, poor water-holding ability, and high concentrations of metals such as Ni, Co, and Cr [7,8,9,10,61], and the relative importance of these factors has been an ongoing point of debate in the literature. The design of our study did not investigate many of these variables and was not intended to resolve this debate. However, the mere fact that accessions of *O. serpyllifolia* from serpentine habitats could be shown to possess substantially higher Ni-tolerance than those from limestone habitats indicates that soil Ni toxicity represents a sufficiently important selective pressure to cause genetic divergence among populations.

Other investigators have used hydroponic common-garden methodology to examine intraspecific variation in Ni tolerance in other *Odontarrhena* species. Two of these studies did not find a positive association between Ni concentrations in soil and Ni tolerance in plants [62,63]; however, these were conducted with the obligate serpentinophytes *O. bertolonii* (Desv.) Jord. & Fourr. (=*Alyssum bertolonii* Desv.) and *O. lesbiaca* Candargy (=*Alyssum lesbiacum* (Candargy) Rech.f.), which were collected across a much narrower range of soil Ni concentrations and showed much less variation in tolerance indices among the accessions. A third example, *O. inflata* (Nyár.) D.A.German (=*Alyssum inflatum* Nyár.), is a facultative hyperaccumulator, but the serpentine and non-serpentine populations were adjacent in a small area, which may have limited opportunities for genetic divergence [64] compared to the more geographically extensive collections investigated here.

In the present study, there were also physiological differences among accessions within a given soil type (Figure 3). For example, growth of limestone accessions L1 and L2 was severely inhibited in the 100 µM Ni treatment, but accession L3 was much more tolerant, producing mean values of root and shoot mass not significantly different from those of some serpentine accessions. In the 300 µM Ni treatment, all limestone accessions showed strong inhibition of growth, but so too did the plants from serpentine site S1, which was significantly more Ni-sensitive than the other serpentine accessions. These finer population-level differences in Ni tolerance were not obviously related to the mean soil Ni concentrations reported in Table 2. However, our soil data were based on limited sampling, with no attempt to comprehensively survey the edaphic variability within each site, nor the solubility and bioavailability of Ni, which can vary greatly depending on the physico-chemical properties of the soil matrix [65]. The wide range of Ni concentrations observed in field-collected leaves from S1 (Table 2) could, for example, imply that some plants were rooted in soils with lower Ni availability.

Genetic factors could also potentially influence variation among plants on the same soil type; for example, the moderately high level of Ni tolerance in accession L3 could result from gene flow from nearby serpentine populations, such as those near site S3, only 30 km away. The intervening region now consists primarily of agricultural and urban land, and the degree of connectivity between the populations would merit further investigation using genetic and biogeographic methods [44,66].

Two reference species were used in this study. *Alyssum montanum* demonstrated very low tolerance of Ni-amended culture solutions, as expected from previous studies [47,48]. In contrast, *Clypeola jonthlaspi* possessed approximately the same degree of Ni tolerance as the limestone accessions of *O. serpyllifolia* (Figure 3, Table 4). Specifically, there were no statistically significant differences, at any solution Ni concentration, between the mean Ni tolerance (expressed as relative root or shoot mass) of *C. jonthlaspi* and accessions L1 and L2 of *O. serpyllifolia*. This surprising finding is discussed in greater detail below.

Uptake and accumulation of elements other than Ni may provide some insight into mechanisms of Ni toxicity and tolerance. In general, serpentine accessions of *O. serpyllifolia* showed a very strong ability to maintain homeostasis of elemental concentrations in both root and leaf tissues even when grown in solutions with elevated Ni concentrations. In contrast, at the same time that relative biomass production was declining and Ni concentrations were increasing in the limestone *O. serpyllifolia*, *C. jonthlaspi*, and *A. montanum* plants, the concentrations of other elements fluctuated widely. As solution Ni concentrations increased, Ca concentration increased in the roots and decreased in the leaves of the less tolerant accessions, suggesting that Ni may interfere with Ca transport from the root to the shoot (e.g., at the xylem loading step) in these plants. Concentrations of Mg and K generally declined with increasing Ni in both root and leaf tissues. Fe concentration showed inconsistent changes in foliar tissues, but a strong tendency to increase in the roots as exogenous Ni concentrations increased. Similar interference with Fe homeostasis has been implicated as a cause of Ni toxicity in *O. inflata* in Iran [67]. It is thus particularly noteworthy that the effects of Ni on root Fe concentrations were less severe in serpentine accessions of *O. serpyllifolia* than in the limestone accessions or reference species.

Principal components analyses of the combined patterns of Ca, Mg, K, and Fe in plant tissues also supported the conclusion that *O. serpyllifolia* plants from serpentine soils possess a superior ability to maintain relatively constant elemental concentrations when exposed to high external Ni concentrations. In addition, these analyses suggested that the species studied here may show distinctive patterns of correlated variation in their elemental profile or “ionome” [68], leading to significant differences in principal component scores. Significant variation was also found in comparisons between limestone and serpentine accessions of *O. serpyllifolia*, suggesting population-specific differentiation in elemental profile; however, as for tolerance and hyperaccumulation, serpentine and limestone accessions exhibited a pattern of continuous variation, with some populations appearing intermediate.

It should be noted that these variations in elemental concentrations were observed in plants growing in a standard 0.1-strength Hoagland solution varying only in the concentration of NiSO_4_. The relative amounts of elements in this solution are intended to mimic those in typical agricultural soils [69]. The trends described here might be different if plants were grown in nutrient solutions approximating the elemental composition of a serpentine soil (e.g., high Mg:Ca ratio), which could be a potential subject for future study. Another recent study of population-level variation in Iberian *O. serpyllifolia*, which was part of a broader study of molecular variation by Quintela-Sabarís et al. [57], compared plants sampled from serpentine and limestone populations grown in a common garden, and found that foliar Mg concentrations were higher in limestone accessions, whereas foliar K and Ca concentrations were higher in serpentine accessions (Fe was not included in their study). The results of the present research are broadly in agreement for Mg and K, but not for Ca. However, there are important methodological differences between the studies. Quintela-Sabarís et al. [57] cultivated plants in a standardized serpentine soil (amended with small additions of compost and perlite), thus imposing the full elemental profile typical of serpentine substrates, in contrast to our study that was designed specifically to manipulate the availability of Ni against a constant background. It is also difficult to compare the bioavailability of metal ions in a soil matrix with those in a more precisely defined and homogeneous nutrient solution, so these two studies are best regarded as providing independent and complementary information on the performance of serpentine and limestone accessions of *O. serpyllifolia*.

In the highest Ni treatment tested (300 µM), foliar Ni concentration in *A. montanum* nominally reached 2.15 g kg^−1^ (Figure 4), exceeding the 1 g kg^−1^ criterion that defines hyperaccumulation [1]. However, in this treatment, the *A. montanum* plants were extremely unhealthy, and in most cases dead, with root and shoot biomass reduced to 4% and 15% of their maximum values, respectively. This is likely an extreme example of “breakthrough accumulation” [11,70], in which metal toxicity and a loss of membrane integrity leads to unregulated uptake of metals and transport to the shoot. Breakthrough should not be regarded as equivalent to hyperaccumulation, because the plants lack sufficient metal tolerance to maintain a viable population in the field. The conclusion that *A. montanum* is not a hyperaccumulator is also supported by its Ni translocation quotient, which was consistently below 1 (Figure 6), whereas hyperaccumulators typically show higher metal concentrations in their leaves than roots [56]. Inability to export Ni from the root tissue by translocation to the shoot may be a factor in the lack of Ni tolerance in this species [46].

Genuine metal hyperaccumulation involves uptake of elements and their accumulation in the shoot above a specified concentration criterion, under conditions that do not adversely affect plant fitness. To examine this relationship in the current dataset, the foliar Ni concentration found in plants at the mNOEC (maximum no observed effect concentration) was compared for the different accessions (Table 4). Using this method and the 1 g kg^−1^ criterion for Ni hyperaccumulation, *A. montanum* would not be classified as a Ni hyperaccumulator, but all accessions of *O. serpyllifolia* would be, as would *C. jonthlaspi*. This is further supported by the observation that both serpentine and limestone accessions of *O. serpyllifolia*, and also *C. jonthlaspi*, had Ni translocation quotients of 1.0 or greater in most treatments (Figure 6). The finding that the ability to hyperaccumulate is a species-wide property in *O. serpyllifolia* is consistent with the conclusion for almost all facultative hyperaccumulators that have been investigated [11].

In this study, *C. jonthlaspi* exhibited sufficient tolerance and translocation ability to accumulate foliar Ni concentrations exceeding 1 g kg^−1^ without significant reduction in growth, thus demonstrating key physiological characteristics of a hyperaccumulator. To date, *Clypeola* species have never been recorded as hyperaccumulators in the field, even in two instances when they were collected on serpentine sites where other hyperaccumulators occur [71,72]. It would be inappropriate to classify this species as a Ni hyperaccumulator based on evidence from hydroponic culture alone. While determining the mNOEC measured under laboratory conditions provides some safeguard against conflating breakthrough with hyperaccumulation, the ability to survive and reproduce while maintaining high foliar metal concentrations under field conditions is an important dimension of the accepted definition of hyperaccumulation [1,70,73]. Further research is needed to understand this seemingly contradictory behavior, which may simply reflect incomplete sampling of this taxon from a sufficiently wide range of sites in the field.

It was noted that, amongst the serpentine populations, accession S1 had significantly lower Ni tolerance than accessions S2 and S3 when grown on 300 µM Ni (Figure 3, Table 4). These plants displayed the highest foliar Ni concentrations of any accession in this study (Figure 4), exceeding 18 g kg^−1^ (i.e., 1.8% of dry leaf mass). This may well be a manifestation of the onset of breakthrough accumulation, given that the root and shoot masses of accession S1 in this treatment were reduced to approximately 40% and 35% of their control values, respectively. If so, this would be, to our knowledge, the first report of plants exhibiting metal tolerance and hyperaccumulation at one exposure concentration, but breakthrough behavior at a higher concentration that exceeds their tolerance limits.

In both the 30 and 100 µM Ni treatments, the limestone accessions of *O. serpyllifolia* had foliar Ni concentrations and translocation quotients significantly higher than those in the serpentine accessions (Figure 5 and Figure 6). Similar trends have been reported for zinc in *Noccaea* (=*Thlaspi*) *caerulescens* [22,74] and *Arabidopsis halleri* [75,76], as well as for cadmium in *N. caerulescens* [51]. The possibility that populations on low-metal soils may possess greater physiological ability to take up and concentrate metals than those on high-metal soils is intriguing in the context of hypotheses that metal hyperaccumulation may provide adaptive benefits for plants [77,78]. However, this conclusion must be treated with caution in the present case. At 100 µM Ni, the limestone accessions were experiencing Ni toxicity, with decreased root and shoot mass, which was not found for the serpentine accessions, so it is possible that the higher foliar Ni concentrations were another manifestation of breakthrough under metal-induced stress. The 30 µM Ni treatment did not cause statistically significant suppression of plant growth in the limestone accessions (Table 4); however, lack of a statistically significant effect at conventionally used error rates (α = 0.05) may be a consequence of insufficient statistical power and does not rule out biologically significant effects that might be detectable by a more sensitive experimental design or larger sample. In the present case, there is a possibility that exposure to 30 µM Ni solution, while causing no significant reduction in plant mass, might elicit more subtle physiological perturbations, including effects on metal homeostasis. Whenever plants with lower tolerance exhibit higher metal uptake and accumulation, the possibility of stress-induced uptake must be considered.

On the other hand, plants from all three limestone accessions showed lower foliar Ni concentrations at 300 µM Ni than at 100 µM, indicating that stressful conditions do not consistently cause high levels of metal accumulation. In this case, the root systems of the plants became very stunted early in the experiment, which may have limited final Ni uptake. Inconsistent performance of plants under extreme stress is not unexpected, but probably has limited ecological relevance. The observation that serpentine accessions of *O. serpyllifolia* had the highest foliar Ni concentrations in the 300 µM Ni treatment (Figure 5) and also the highest translocation quotient in this treatment (Figure 6) is probably a direct result of their Ni tolerance, allowing them to remain physiologically active in a high-Ni environment. Metal tolerance and metal hyperaccumulation are at least partially independent characters [21,22,23], but they interact with each other in complex ways, and a high degree of cellular metal tolerance will be a prerequisite for sustained metal accumulation [13]. In Ni-hyperaccumulating species of *Odontarrhena*, free histidine plays an important role in detoxifying Ni in the root and facilitating its translocation to the shoot [47]. Moreover, it has been shown that root histidine concentrations in the facultative hyperaccumulator *O. serpyllifolia* are intermediate between those of the non-accumulator *Alyssum montanum* and the obligate hyperaccumulator *O. lesbiaca* [48], suggesting that this trait might be closely linked to adaptation of the hyperaccumulator species of *Odontarrhena* to serpentine soils.

Experiments using hydroponic methodology have certain advantages compared to those involving cultivation in soil, including precise control over the concentrations of elements. Binding or adsorption of ions to a soil matrix may cause unpredictable effects on the availability of elements to plants, and it may be more difficult to achieve uniform treatment conditions. On the other hand, solution culture may not accurately mimic the processes that occur when plants grow in metalliferous soils, especially those involving the effects of root exudates on rhizosphere soils and the effects of the plant microbiome, including bacterial and fungal endophytes, mycorrhizae, and other rhizosphere microorganisms [79,80].

Although the present study was not designed to include a formal taxonomic investigation of *O. serpyllifolia*, its conclusions may inform current taxonomic debates about the status of this taxon. As mentioned earlier, some workers have proposed segregation or “splitting” of *O. serpyllifolia* in order to classify plants from serpentine soils as distinct subspecies or even species. Although a few subtle morphological differences between populations were noted in the published taxonomic descriptions [39,40], it appears that a crucial factor in naming these distinct serpentine-endemic taxa was the notion that hyperaccumulator physiology is a distinctive and discrete feature of plants growing on serpentine [33,58,59]. In the context of more recent genetic and karyological analyses [44,81] that argue against taxonomic segregation of the serpentine populations of *O. serpyllifolia*, our results serve to highlight the wide range of physiological tolerances that are encompassed within a single species.

At a higher taxonomic level, the patterns described here involving ecophysiology of Ni tolerance and accumulation are remarkably congruent with recent phylogenetic treatments [27,28,29,30,31]. *Odontarrhena* and *Clypeola* have been resolved as very closely related genera [31], with a putative sister-group relationship, and our findings suggest they possess physiological similarities, especially when comparing *C. jonthlaspi* to the limestone accessions of *O. serpyllifolia*. As the one representative of *Alyssum* (*sensu stricto*) included in our study, *A. montanum* showed quite different physiological behavior, in keeping with its more distant phylogenetic placement. The remarkable diversity of Ni hyperaccumulator species in *Odontarrhena* has been and will undoubtedly remain a fertile subject for research, but broader experimental testing of species in *Clypeola*, *Alyssum*, and related genera is needed to elucidate the phylogenetic distribution of potential Ni tolerance and hyperaccumulation, and their evolution within the Alysseae.

## 4. Materials and Methods

### 4.1. Sample Collection

Field sites for collection of *O. serpyllifolia* were located based on herbarium records, past publications, and the advice of local specialists. Three serpentine sites were chosen to represent the three main ultramafic complexes in the Iberian Peninsula, along with three sites on limestone across a similar geographic range. The resulting accession localities are described in the Results (Figure 1, Table 1).

To confirm the hyperaccumulator status of plants in the field at the time of collection, leaves were crushed in filter paper impregnated with dimethylglyoxime, which displays a pink color in the presence of high concentrations of Ni. Additional leaf samples were taken from four plants at each site, dried, and stored in paper envelopes until analysis. From each site, a bulk collection of ripe fruits was made from at least 10 widely spaced source plants. Fruits were crushed by hand and seeds were carefully separated from the pericarps and other inflorescence debris. Samples of soil were also collected from the uppermost 15 cm of the soil profile. At least 3 soil samples were collected at each site, with additional samples taken in cases where the plants were distributed patchily over a large area.

Seeds of *Clypeola jonthlaspi* var. *jonthlaspi* (serial no. 413532), originally collected from a population in Jordan, were obtained from the Millennium Seed Bank (Royal Botanic Gardens, Kew, U.K.) and planted in potting compost in a glasshouse (see details below). The plants fruited abundantly after about 3 months, presumably via self-fertilization as no effort was made to facilitate cross-pollination, and the seeds for our experiments were the progeny of this process. The one-seeded fruits of *C. jonthlaspi* function as propagules, so they were germinated without removing the pericarp.

Seeds of *Alyssum montanum* were purchased from a commercial garden supply company (Dobies of Devon, Paignton, Devon, UK). In the wild, *A. montanum* is part of a complex of related species, including both diploid and polyploid races, with a history of varied taxonomic treatments [46,82,83,84]. The horticultural seeds we purchased included no information to document the provenance of the material.

### 4.2. Germination and Hydroponic Cultivation

Plants were cultivated in a glasshouse with natural solar irradiation supplemented with sodium-vapor lamps to provide 16 h day-length, and with minimum temperatures maintained at 25 °C in the daytime and 14 °C at night. Seeds were germinated on the surface of horticultural sharp sand moistened with deionized water. Seeds from each accession were sown onto sand in a separate tray, under a transparent dome to maintain high humidity. Seedlings were allowed to grow on the sand until the hypocotyl was at least 1 cm long (approximately 17 d) before transfer to hydroponic solution culture.

Hydroponic cultivation was conducted in 1.2 L polycarbonate boxes coated on their exterior surface with opaque paint to keep the interior dark and thus minimize algal growth. The top of each box was drilled with 10 holes, each 6–8 mm in diameter. Seedlings were individually inserted into holes and held in place with a small piece of soft polyurethane foam. Each box received 10 seedlings from a single accession (i.e., a single source site of *O. serpyllifolia*, or one of the reference species, *C. jonthlaspi* or *A. montanum*). A total of 13 boxes were set up for each accession.

The nutrient solution was based on that used by Roosens et al. [51], intended to mimic the generally low nutrient status of metalliferous soils, and consisted of modified 0.1-strength Hoagland solution macronutrients [69] containing 0.5 mM KNO_3_, 0.1 mM KH_2_PO_4_, 0.4 mM Ca(NO_3_)_2_, and 0.2 mM MgSO_4_, together with modified 0.2-strength Long Ashton micronutrients [85] containing 20 μM Fe-EDDHA (ferric ethylenediamine-di-(2-hydroxyphenylacaetate): Duchefa Biochemie, Haarlem, The Netherlands), 10 μM H_3_BO_3_, 2.0 μM MnSO_4_, 0.2 μM CuSO_4_, 0.2 μM ZnSO_4_, and 0.1 μM NaMoO_4_. For 21 d, seedlings were allowed to acclimatize to hydroponics, with no added Ni in the medium. Solutions were changed once during this period. No additional aeration was used.

After the 21 d pre-treatment period, hydroponic media were replaced with Hoagland solution incorporating NiSO_4_ at defined concentrations. Any plants that died during pre-treatment were removed at this time and omitted from the dataset. Because of the possibility of nickel deficiency in long-term cultivation [86], we did not use a nickel-free solution as our experimental control and instead employed a micronutrient concentration of 0.1 μM Ni in the control solution. The remaining treatment concentrations were 10, 30, 100, and 300 μM Ni. Solutions were changed weekly for the duration of the experiment.

It was not possible to employ a perfectly balanced experimental design because of limitations imposed by the numbers of viable seedlings. Thus, for the non-serpentine accessions L1, L2, and L3, and the reference species *Clypeola jonthlaspi* (Cj) and *Alyssum montanum* (Am), the 13 boxes were allocated as 3 boxes each at NiSO_4_ concentrations of 0.1, 10, 30, and 100 μM, plus 1 box at 300 μM. We reduced the replication at 300 μM because we expected little survival of these non-serpentine accessions at the highest Ni concentration. For the serpentine accessions S1, S2, and S3, the 13 boxes were allocated as 3 boxes each at NiSO_4_ concentrations of 0.1, 30, 100, and 300 μM, plus 1 box at 10 μM. This scheme was employed because we expected no measurable effects of 10 μM NiSO_4_ on growth of the serpentine accessions.

After 8 weeks of cultivation on Ni-amended media, all plants were harvested. Each shoot was cut off at its base, rinsed in a solution of 1% (*w*/*v*) sodium dodecyl sulfate (SDS) plus 5 mM ethylenediaminetetraacetic acid (EDTA) to desorb surface metal ions, and transferred to an individual paper envelope. The roots of all the plants in a given hydroponic box were often inseparably intertwined, so all the roots in a box were harvested *en masse*, rinsed as above, blotted dry on paper towels, and placed in envelopes to dry.

### 4.3. Sample Preparation and Analysis

Shoots and roots were dried at 80 °C in a fan-assisted oven for at least 72 h. Dry matter was weighed to determine the shoot mass per plant and the root mass per box. Samples of leaves and roots were collected for Ni analysis. Ideally, samples weighed 0.05–0.10 g; however, smaller leaf samples were used for some of the smallest plants. All masses were recorded to four decimal places.

Samples were digested in 4 mL of 15 M HNO_3_ for 16 h at 20 °C. Each digest was then diluted with 10 mL of deionized water and filtered to remove tissue fragments. Determination of Ni, Ca, Mg, Fe, and K in leaf and root digests was conducted using an atomic absorption spectrophotometer with an air–acetylene flame, a double-beam optical system, and deuterium arc background correction (AAnalyst 100; Perkin-Elmer, Beaconsfield, Buckinghamshire, UK). Prior to Ca analysis, LaCl_3_ was added at a concentration of 1% (*w*/*v*) to suppress interference, as per standard instrumental protocols.

Field-collected leaf samples were washed, dried, and digested in an identical manner to materials from the hydroponic experiments. Dried soil samples, sieved through a 1 mm nylon mesh, were weighed out to a mass of 1.0 g, then digested in 3 mL of 15 M HNO_3_ for 4 h at 90 °C in Pyrex tubes in a block heater, digested again in 12 M HCl at for 2 h at 80 °C in the block heater, and finally filtered and diluted to 50 mL using deionized water. Concentrations of Ni in field-collected leaf and soil digests were measured using atomic absorption spectrophotometry as for the hydroponic samples.

### 4.4. Data Analysis

Shoot mass and elemental concentrations were averaged across the plants in a given box, and the box mean was used as the basic unit for further statistical analysis. This avoided pseudoreplication, and also made the levels of replication for shoot data equivalent to those for roots, which were harvested on a box rather than individual basis. The total root mass per box was divided by the number of surviving plants, which varied due to mortality during the pre-treatment phase of cultivation, to derive mean root mass per plant.

A Shapiro–Wilk test indicated that plant mass and Ni concentration did not deviate significantly from a normal distribution, and a Brown–Forsyth test indicated that sample variances for these characteristics did not differ significantly between plant groups and treatment levels, thus satisfying the assumptions of parametric statistical procedures. Analysis of variance (ANOVA) and multiple-comparison procedures (Dunnett’s test or Tukey’s HSD test, as appropriate) were conducted to compare accessions and treatments. All statistical tests were performed using JMP 11.2.0 software (©2013, SAS Institute, Cary, NC, USA).

## 5. Conclusions

1. Accessions of *Odontarrhena serpyllifolia* from serpentine soil were substantially more Ni-tolerant than those from limestone soil; however, intermediate degrees of tolerance exist on both soil types, suggesting a spectrum of continuous variation in this character.
2. All accessions of *O. serpyllifolia*, whether from serpentine or limestone, were capable of hyperaccumulating Ni in hydroponic cultivation. There was variation among accessions in ability to hyperaccumulate, but once again, it was continuous rather than discrete.
3. Hyperaccumulators are best defined based on metal concentrations in natural self-sustaining populations. In laboratory studies, we propose that physiological ability to hyperaccumulate can be tentatively identified based on foliar metal concentrations exceeding a threshold criterion in plants cultivated in substrates with metal availability at or below the maximum No Observed Effect Concentration (mNOEC), thus providing a safeguard against artifactual “breakthrough” of metals in plants experiencing metal toxicity.
4. Accessions of *O. serpyllifolia* from serpentine substrates maintained relatively constant concentrations of essential nutrient elements in their tissues when grown in a range of Ni concentrations, whereas those from limestone substrates showed an inability to maintain elemental homeostasis when grown in elevated Ni.
5. *Clypeola jonthlaspi*, closely phylogenetically related to *Odontarrhena*, showed some physiological characteristics of hyperaccumulators, although it has not been observed to hyperaccumulate Ni in nature. Further studies of this species are needed.

## Figures and Tables

**Table 1:** Source information for seeds. Accession codes beginning with S indicate *Odontarrhena serpyllifolia* from serpentine outcrops, whereas those beginning with L represent the same species from limestone areas. Locations of field collections are shown in Figure 1. Code Cj represents *Clypeola jonthlaspi* and Am represents *Alyssum montanum*, both of which were grown from commercially sourced seeds.

Accession Code	Substrate	Source	Latitude/Longitude	Year Collected
S1	Serpentine	Barazón (Galicia, Spain)	42°51.058′ N, 8°00.313′ W	2011
S2	Serpentine	Samil (Bragança, Portugal)	41°46.774′ N, 6°45.106′ W	1999
S3	Serpentine	Carratraca (Andalucía, Spain)	36°51.223′ N, 4°48.736′ W	2011
L1	Limestone	Rubiá (Galicia, Spain)	42°29.260′ N, 6°49.830′ W	2011
L2	Limestone	Morata de Tajuña (Madrid, Spain)	40°14.997′ N, 3°29.071′ W	2002
L3	Limestone	Alhaurín de la Torre (Andalucía, Spain)	36°38.780′ N, 4°36.077′ W	2002
Cj	N/A	Millennium Seed Bank (Origin: Jordan)	N/A	2011 *
Am	N/A	Purchased from Dobies of Devon, UK	N/A	2012 *

* Date of purchase.

**Table 2:** Nickel concentration in soils and in field-collected foliage of *Odontarrhena serpyllifolia* from sites in Spain and Portugal. Initial letter of accession code indicates geological substrate (S = serpentine; L = limestone). Field sites are described in Table 1. The number of soil samples collected per site (*N*) varied depending on the area and heterogeneity of the habitat. Leaf Ni concentrations exceeding 1 g kg^−1^ of dry biomass are regarded as hyperaccumulation.

Accession Code	Soil Ni (g kg^−1^)			Leaf Ni (g kg^−1^)		
	*N*	Mean ± SE	Range	*N*	Mean ± SE	Range
S1	3	3.614 ± 0.781	2.604–5.150	4	4.595 ± 2.681	0.165–11.189
S2	12	2.962 ± 0.102	2.550–3.841	4	1.377 ± 1.236	0.104–5.084
S3	6	1.593 ± 0.205	1.075–2.426	4	2.513 ± 0.236	2.067–3.127
L1	3	0.028 ± 0.004	0.020–0.034	4	0.028 ± 0.003	0.022–0.035
L2	3	0.002 ± 0.001	0.002–0.003	4	0.025 ± 0.006	0.013–0.041
L3	5	0.007 ± 0.003	0.000–0.017	4	0.007 ± 0.003	0.001–0.014

**Table 3:** Summary of *F* ratios from ANOVA on differences in mean root mass and shoot mass of plants, in response to Ni amendments of hydroponic media at concentrations of 0.1, 10, 30, 100, and 300 µM. ANOVA was conducted separately for each of the six accessions of *Odontarrhena serpyllifolia* (S1–S3 from serpentine sites; L1–L3 from limestone sites) and the two reference species, *Clypeola jonthlaspi* (Cj) and *Alyssum montanum* (Am). *F* ratios in bold font represent statistically significant differences (* *p* < 0.05; *** *p* < 0.001); normal font indicates no significant difference (*p* > 0.05). In each ANOVA, treatment df = 4 and error df = 8.

Accession	Root Mass	Shoot Mass
S1	7.008 *	6.825 *
S2	1.034	2.296
S3	0.777	0.194
L1	109.393 ***	91.234 ***
L2	32.409 ***	39.239 ***
L3	28.557 ***	62.427 ***
Cj	45.873 ***	38.514 ***
Am	30.350 ***	57.051 ***

**Table 4:** The maximum no observed effect concentration (mNOEC) based on Dunnett’s multiple comparison procedure, for six accessions of *Odontarrhena serpyllifolia* from either serpentine (S1–S3) or limestone (L1–L3) soil, plus *Clypeola jonthlaspi* (Cj) and *Alyssum montanum* (Am). Plants were grown in hydroponic growth media containing Ni at concentrations of 0.1, 10, 30, 100, and 300 µM. The mNOEC is the highest concentration that produced no statistically significant reduction in growth, as indicated by shoot or root mass, when compared to growth in the control (0.1 µM) concentration. The last column shows the foliar Ni concentration of the plants at their mNOEC.

Accession	mNOEC (µM) Based on Shoot Mass	mNOEC (µM) Based on Root Mass	Foliar [Ni] (g kg^−1^) at mNOEC
S1	100	100	6.14
S2	≥300	≥300	11.52
S3	≥300	≥300	11.68
L1	10	30	2.54 *
L2	30	30	2.97
L3	30	30	2.21
Cj	30	30	2.78
Am	0.1	0.1	0.001

* Assuming mNOEC = 30 µM based on root mass; at 10 µM, the foliar [Ni] of accession L1 was 0.825 g kg^−1^.

**Table 5:** Summary of *F* ratios from ANOVA of differences in mean concentrations of Ca, Mg, K, and Fe in plant tissues in response to Ni amendments of hydroponic growth solutions at concentrations of 0.1, 10, 30, 100, and 300 µM. Plants have been grouped into four categories: *Odontarrhena serpyllifolia* from serpentine soil (Os-S), *O. serpyllifolia* from limestone soil (Os-L), *Clypeola jonthlaspi* (Cj), and *Alyssum montanum* (Am); leaves and roots were analyzed separately. *F* ratios in bold font represent statistically significant differences (* *p* < 0.05; ** *p* < 0.01; *** *p* < 0.001); normal font indicates no significant difference (*p* > 0.05). In all ANOVAs, treatment df = 4; for *O. serpyllifolia* accessions, error df = 34; for *C. jonthlaspi* and *A. montanum*, error df = 8.

Accession Group		Concentration of Element in Plant Tissue			
	Tissue	Ca	Mg	K	Fe
Os-S	Leaf	0.59	0.95	1.88	0.49
Os-L	Leaf	4.28 **	6.50 ***	2.05	15.39 ***
Cj	Leaf	13.70 **	8.94 **	17.69 ***	4.92 *
Am	Leaf	4.88 *	5.01 *	1.87	2.20
Os-S	Root	2.23	7.68 ***	3.17	51.68 ***
Os-L	Root	16.59 ***	4.54 **	5.73 **	56.54 ***
Cj	Root	31.71 ***	25.65 ***	15.25 ***	475.24 ***
Am	Root	15.84 ***	3.59	5.94 *	9.36 **

**Table 6:** Summary of *F* ratios from ANOVA of principal components analysis (PCA) scores, in response to nickel amendments of hydroponic growth solutions at concentrations of 0.1, 10, 30, 100, and 300 µM. Plants have been grouped into four categories: *Odontarrhena serpyllifolia* from serpentine soil (Os-S), *O. serpyllifolia* from limestone soil (Os-L), *Clypeola jonthlaspi* (Cj), and *Alyssum montanum* (Am); leaves and roots were analyzed separately. *F* ratios in bold font represent statistically significant differences (* *p <* 0.05; ** *p <* 0.01; *** *p <* 0.001); normal font indicates no significant difference (*p >* 0.05). In all ANOVAs, treatment df = 4; for *O. serpyllifolia* accessions, error df = 34; for *C. jonthlaspi* and *A. montanum*, error df = 8.

Accession Group		Principal Component Axis	
	Tissue	PCA1	PCA2
Os-S	Leaf	1.00	1.11
Os-L	Leaf	5.97 ***	13.25 ***
Cj	Leaf	15.74 ***	6.79 *
Am	Leaf	4.89 *	2.19
Os-S	Root	2.28	6.31 ***
Os-L	Root	28.30 ***	2.80 *
Cj	Root	242.04 ***	9.61 **
Am	Root	9.35 **	2.17

## Data Availability

Not applicable.
